# A wearable fetal movement detection system for pregnant women

**DOI:** 10.3389/fmed.2023.1160373

**Published:** 2023-07-24

**Authors:** Manping Qin, Yong Xu, Yubao Liang, Tie Sun

**Affiliations:** ^1^School of Artificial Intelligence and Smart Manufacturing, Hechi University, Hechi, China; ^2^School of Automation and Electrical Engineering, University of Science and Technology Beijing, Beijing, China

**Keywords:** fetal movement detection, wearable devices, Cortex-M4, accelerometer, Bluetooth

## Abstract

A wearable device-based fetal movement detection system for pregnant women is proposed to resolve the problems of low accuracy of fetal movement detection by fetal heart monitor, difficulties of fetal movement monitoring by pregnant women in person, and inability to monitor for long periods of time by ultrasonic Doppler imaging device. The overall software design flow of the system is proposed after determining the overall structure of the system based on symmetric sensor. The application circuit of the three-axis acceleration sensor MC3672 and its supporting sensor data collection program are designed, and the application circuit of the main control chip NRF52840 with Cortex-M4 core is analyzed. The function of data collection and algorithm recognition result transfer to a smartphone is realized through the fetal movement recognition and algorithm design and Bluetooth communication design. Finally, the system test scheme is introduced, which involves performing functional tests on four healthy pregnant volunteers and analyzing the results. The experimental results show that the average recognition rate and correct rate of this system to recognize fetal movement is 89.74% when using the real fetal movement actively perceived by pregnant women as the standard, achieving a domestic and wearable design of fetal movement monitoring device for pregnant women that can be used to analyze and predict the fetal health condition.

## 1. Introduction

With the increase in the average age of women at conception and the full implementation of China’s three-child policy (1), the number of pregnant women is increasing, particularly among older pregnant women and high-risk mothers. Simultaneously, people’s perceptions of marriage and childbirth have improved, and the concept of eugenics has been emphasized, and an increasing number of pregnant families are concerned about real-time fetal health monitoring, and every family wishes for a healthy baby. In the middle and late stages of pregnancy, fetal movement is an important vital sign of the health of the fetus developing in the mother’s body (2–4), and studies have shown that the number of fetal movements in the uterus of pregnant women can last for days or even weeks before decreasing and disappearing (5–7), so fetal movement detection can detect fetal abnormalities.

At the moment, domestic and foreign researchers have gradually increased their research and exploration in the field of fetal movement signal recognition, particularly in theoretical and algorithmic research, and the related research results are rich. For example, Ge Gaofa introduced adaptive filters of wavelet transform into fetal movement signal processing (8). The literature (4, 9) used time-domain analysis to identify fetal movement and divided it hierarchically. The literature (10) used dynamic time regularization, spectral clustering, and non-negative matrix decomposition techniques to create an algorithm that can detect fetal movements automatically. The majority of domestic and foreign fetal movement detection research and applications are aimed at theoretical design, algorithm, and simulation, and there are few cases of practical, feasible, and wearable fetal movement detection solutions, for example, Ju Yinhui et al. (11) used pressure sensors to acquire fetal movement signals. However, only a single dimensional fetal movement signal could be obtained, and the entire fetal movement signal could not be obtained, posing many limitations for sampling the mother. Wen Hongyu acquired fetal movement signals using an ultrasonic Doppler sampling technique (12), which can only be used in specific scenarios and cannot be used for an extended period of time.

Furthermore, the market’s common home Doppler type fetal heart monitor and home stethoscope type fetal heart monitor are primarily for fetal heart monitoring, and the accompanying fetal movement detection function is poor and inaccurate. Meanwhile, because traditional fetal movement detection methods such as counting fetal movement by the pregnant woman herself and ultrasonic Doppler imaging devices cannot monitor fetal movement for an extended period of time, research into wearable device-based fetal movement detection systems for pregnant women and their associated equipment is critical.

## 2. A wearable fetal movement detection system

### 2.1. The overall design of wearable fetal movement detection system

**Figure 1:**
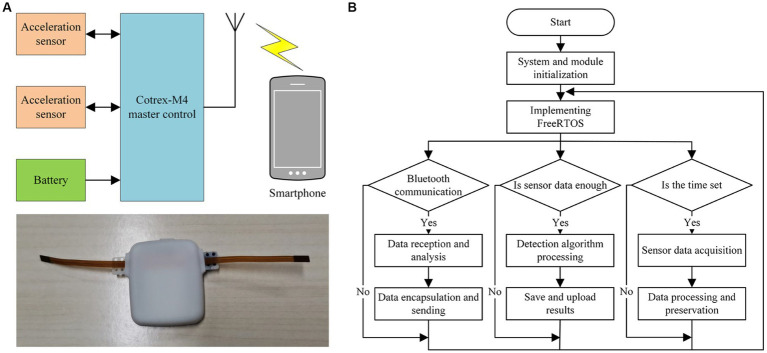
Block diagram of the wearable fetal movement detection system: **(A)** The structure and prototype object diagram. **(B)** Overall software design flow chart.

Figure 1A depicts the structure and prototype of the wearable pregnant woman fetal movement detection system, which includes two acceleration sensors, a Cortex-M4 main control chip, battery management, a Bluetooth antenna, a smartphone, and other functional modules. The embedded hardware part of the system employs the Nordic Semiconductor NRF52840 as the control core, which is a microprocessor chip with an integrated Bluetooth module that is responsible for analyzing and processing the data collected by the acceleration sensors.

The front-end data collection device uses a low-power, low-noise three-axis acceleration sensor MC3672, through low-power loss of Bluetooth wireless communication, and Android smartphone’s real-time information interaction, to achieve the storage and visualization of fetal movement detection data. The front-end data collection device stores and visualizes fetal movement detection data using a low-power, low-noise three-axis acceleration sensor MC3672, Bluetooth wireless communication with low-power loss, and real-time information interaction with an Android smartphone. A 3.7 V rechargeable lithium battery is used for the step-down power supply in the system.

This fetal movement detection system’s software is based on the Cortex-M4 embedded core controller and employs the Keil uVision5 development tool as well as the real-time operating system FreeRTOS (13). FreeRTOS has a smaller memory footprint, lower computational complexity, and the ability to make task calls to multiple sub-threaded tasks at the same time. Figure 1B depicts the overall software design flow of the system. When the processor is turned on, it first initializes the FreeRTOS, acceleration sensor, and power management modules, and then the system scheduler schedules high tasks based on task priority, with the tasks with the highest priority receiving CPU resources first.

The system software function tasks are divided into three parts based on the system function: sensor data collection, fetal movement recognition algorithm processing, and Bluetooth data communication. The acceleration sensor data collection task is given the highest priority, followed by the Bluetooth data communication task, and the fetal movement recognition algorithm processing task is given the lowest priority. When the amount of collected data reaches the threshold value, the system scheduling switches the sensor data collection task and the algorithm recognition task to perform the algorithm recognition processing on the sensor collected data, and the Bluetooth data communication task is in charge of transmitting the sensor collected data and the algorithm recognition result to the smartphone terminal.

### 2.2. Acceleration sensor data collection unit

**Figure 2:**
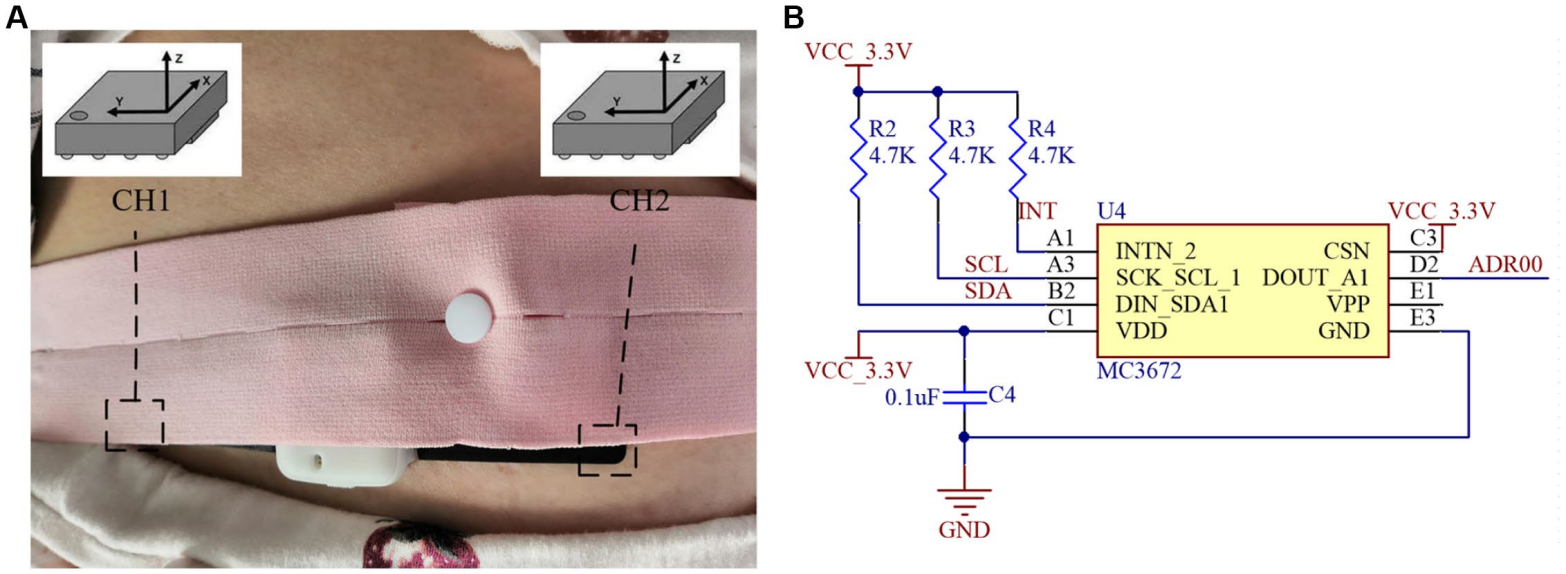
Acceleration sensor data collection unit: **(A)** Collection schematic. **(B)** Application circuit diagram.

Figure 2A illustrates the acceleration sensor data collection schematic, and the MC3672, an acceleration sensor in the WLCSP-8 package from MEMSIC Semiconductor, is used to collect the fetal movement signal (14). This sensor is the smallest three-axis accelerometer with 32 sampling buffers, ultra-low power consumption, high sensitivity, low noise, and integrated digital output, with volumes of 1.29 mm × 1.09 mm × 0.74 mm. As shown in Figure 2B, an MC3672 accelerometer application circuit diagram. The MC3672 sensor’s clock line SCK SCL 1 and data transmission line DIN SDA1 are connected to the NRF52840 master chip’s SCL (P1.00) and SDA (P0.22) pins. The IIC communication protocol is used for data transmission with the master chip, which sends the collected acceleration values on the X, Y, and Z axes to the master control for processing at a rate of up to 1 MHz.

The sensor’s data communication mode uses one host and two slave modes, which means that the master controller and two acceleration sensors communicate with each other via the IIC protocol. When the sensor data collection task is carried out after the FreeRTOS system task scheduling, the host controller first initializes and handshakes with the accelerometer and sets the sensor detection sensitivity 4,096 times/g (1 g = 9.8 m/s^2^) and detection range 2 g. Following that, the host controller begins reading the accelerometer data cyclically via the software timer task, with a sampling frequency of 100 Hz. The acceleration sensor has three axes, each of which corresponds to a register group, and each register group has two registers, one of which stores the high 8 bits of data and the other the low 8 bits of data, and the registers are sequential to each other’s address.

This fetal movement detection system reads the three axes data from the accelerometer registers, uses the 256 high and low 8-bit data of the three axes as an analysis window, merges the 256 high and low 8-bit data for high and low 8-bit processing, saves the conversion to get three groups of 256 16-bit data, and sends it to the algorithm processing and fetal movement recognition tasks. Because the *Z*-axis of the accelerometer placed horizontally will be affected by one gravitational acceleration, the influence of the DC component should be eliminated after system initialization or the user’s mobile phone sends the calibration operation, employing multiple measurements to take the mean value.

### 2.3. Application design of master control chip

**Figure 3:**
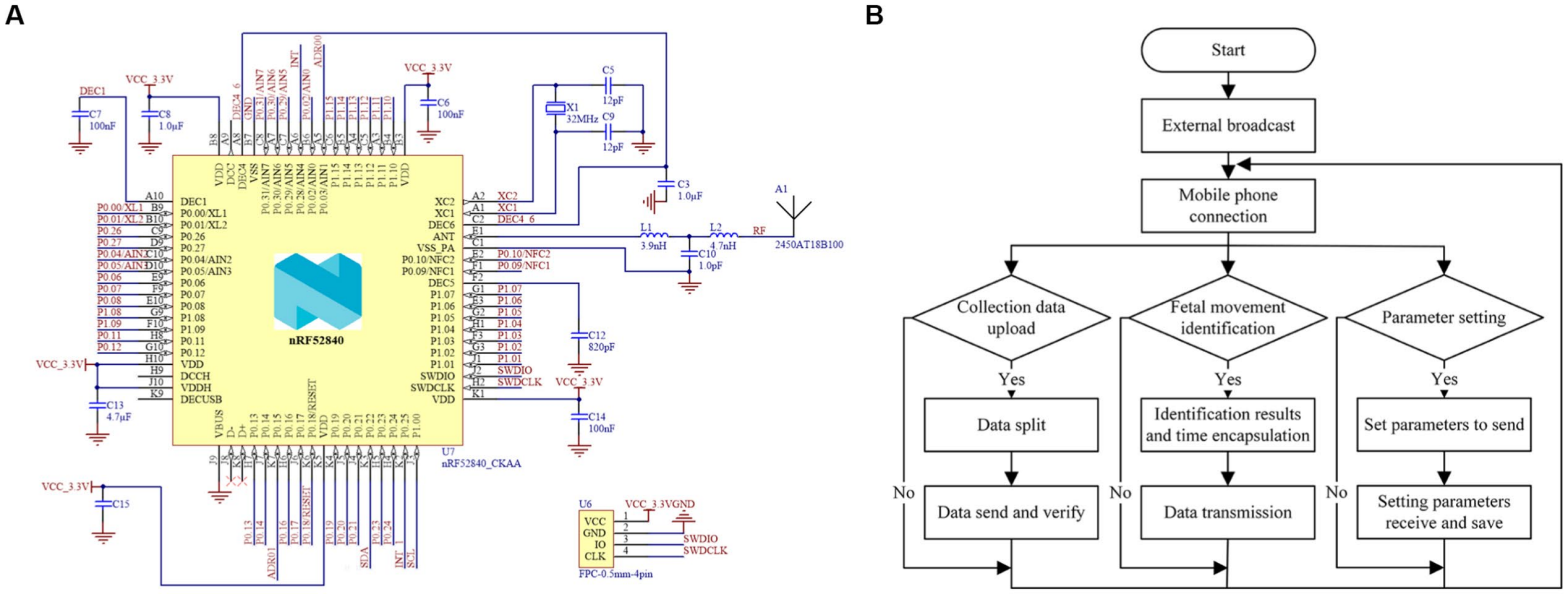
Application design of master control chip: **(A)** Main control chip application circuit diagram. **(B)** Bluetooth data communication flow chart.

The NRF52840 with a floating point unit, up to 64 MHz main frequency, multi-wireless protocol support, and Cortex-M4 core is used as the core controller in the fetal movement detection system (15), and the master control chip application circuit is shown in Figure 3A. A power-on reset circuit, crystal oscillation circuit, Bluetooth antenna matching circuit, power supply interface, and SWD download debug interface are among the peripherals of the main controller application circuit. A1 is the onboard Bluetooth antenna, crystal X1, and capacitors C5 and C9 comprise the master chip’s 32MHZ master clock circuit. Furthermore, because the NRF52840 main control chip includes a low-power Bluetooth 5.2 module, it is necessary to add bypass capacitors at the chip power supply and other pins, such as C6, C7, C8, C12, C14, C15, and so on, to eliminate the impact of high-frequency noise on other circuits and improve the smooth characteristics of chip operation.

This system’s Bluetooth data communication is based on the FreeRTOS system with an integrated Bluetooth protocol stack, and its tasks include uploading data collected by acceleration sensors, outputting effective fetal motion recognition results, and setting parameters for wearable embedded devices, as illustrated in Figure 3B. After being packaged and processed by the system, the Bluetooth data to be sent is transmitted by the data-sending function to the Bluetooth module’s data-transmitting area on the controller chip. After digital-to-analog conversion, the processor in the transmitting area turns on the RF module, the device makes an external broadcast, and the data packets are sent to the data interaction channel to be received by the monitoring software on the smartphone side. The fetal movement recognition result and the original data acquired by the front-end acceleration sensor are uploaded to the smartphone APP side for data storage and visualization via Bluetooth data communication. Furthermore, because the system only has one master clock and the Bluetooth protocol stack operation requires a low-speed protocol stack clock, this study achieves the device Bluetooth module clock requirements through the master chip configuration, from the high-speed clock synthesis of a low-speed clock.

## 3. Fetal movement recognition and algorithm design unit

**Figure 4:**
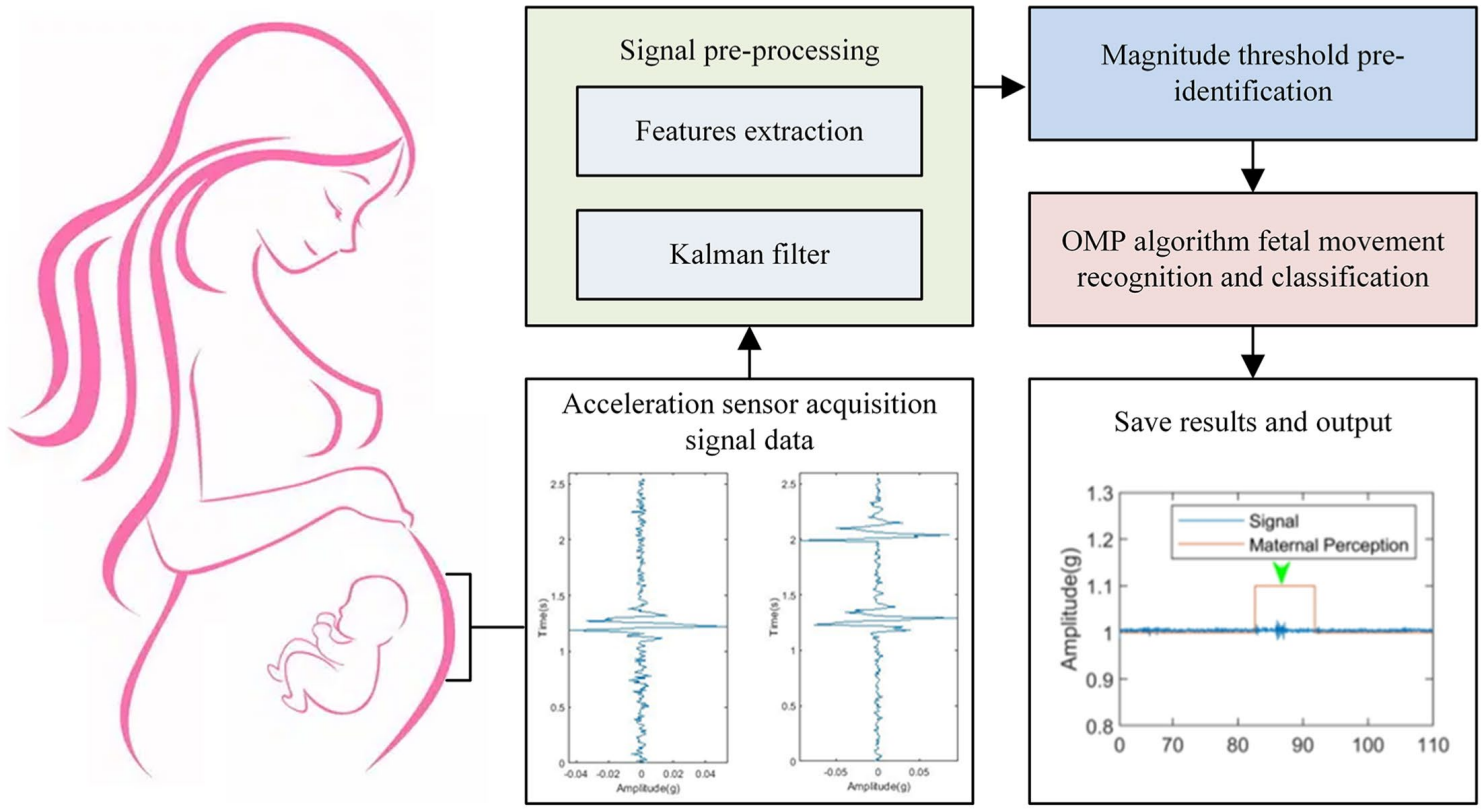
Fetal movement recognition flow chart.

When the amount of data collected by the three-axis acceleration sensors reaches the 256 data per axis threshold, the sensor data collection task and the algorithm recognition task switch tasks under system scheduling to perform algorithm recognition processing on the sensors’ sampled data. The processing task of the fetal movement recognition algorithm is primarily to process the six-axis 256 16-bit data collected and processed by the two three-axis acceleration sensors, as shown in Figure 4.

Input acceleration sensor data collection, signal pre-processing, amplitude threshold pre-recognition, algorithm recognition of fetal movement and fusion classification by Orthogonal Matching Pursuit (OMP) algorithm, saving results and output, and so on are all steps in fetal movement recognition. To begin, the signal pre-processing stage employs the Kalman filtering algorithm to remove some of the interference noise introduced by large maternal movements. The pre-processed data is then processed by the amplitude threshold pre-recognition stage, which filters out artifact signals that are similar to the fetal movement signal. The OMP algorithm is then used to identify and categorize the data. Finally, the result of the fetal movement recognition processing is saved and output to the Bluetooth data communication task while the data upload task is scheduled.

### 3.1. Kalman filtering pre-processing unit

**Figure 5:**
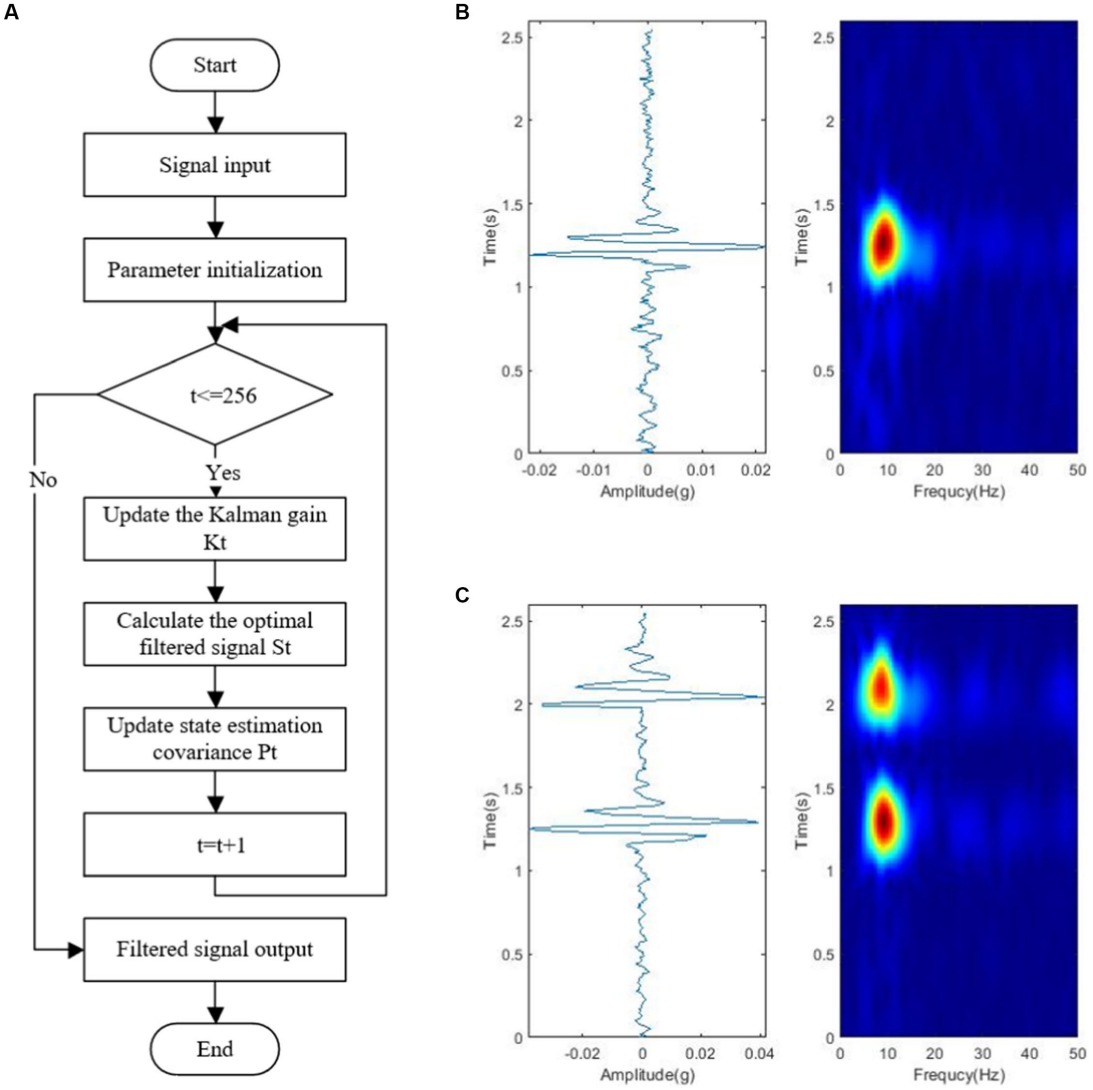
Fetal movement signal map after Kalman filter pre-processing: **(A)** Kalman filter pre-processing flow chart. **(B)** Single peak time domain and time-frequency domain. **(C)** Double peak time domain and time-frequency domain.

The Kalman filter algorithm with low computational effort and high efficiency is used in the pre-processing of a fetal movement signal to solve the problem of the spectrum of fetal movement signal and random interference noise overlapping. The Kalman filter algorithm is based on the estimated value of the previous period and the observed value of the current period to make the best estimate of the current system state. The algorithm implementation primarily consists of prediction and update, as shown in Figure 5A.

Where the original fetal signal x is the input preprocessed signal and the output filtered signal is S The initialized parameters include $S_{1}=x_{0}$ , the initial sampling moment *t* = 1, the state error covariance moment Q, the observation error covariance moment R, and the initial state estimation covariance $P_{0}$ . The Kalman gain $K_{t}$ at a certain t sampling moment is determined by $P_{t-1}$ and R at the t-1 moment, and its expression is Equation (1) and Equation (2).


(1) $$K_{t}=P_{t-1}/\left(P_{t-1}+R\right)$$

(2) $$S_{t}=S_{t-1}+K_{t}\left(x_{t}-S_{t-1}\right)$$

From Equation (2), $S_{t}$ can be calculated from $\;S_{t-1}$ , $K_{t}$ and $x_{t}$ , and in addition, $P_{t}$ can be updated from $P_{t-1}$ , $K_{t}$ and Q through Equation (3).


(3) $$P_{t}=P_{t-1}-K_{t}P_{t-1}+Q$$

Figures 5B,C depicts the Kalman algorithm filtering test results after predicting and correcting the algorithm parameters. The signal burr is reduced after the Kalman algorithm filtering process, and the signal is imaged more smoothly on the time-frequency image, improving the signal-to-noise ratio of the useful fetal movement signal and facilitating the restoration of the low-amplitude fetal movement signal from the noisy background and further signal processing. Furthermore, the maternal body and the sensor may produce artifacts in the acquired raw signal that are very similar to the real fetal movement signal, which the filter cannot completely remove.

### 3.2. Amplitude threshold pre-identification unit

Signal pre-processing can remove interference noise caused by some large maternal movements, but it cannot remove artifactual signals generated by the mother that are very similar to the true fetal movement signal. The amplitude range of the real fetal movement signal is generally between 0.015 g and 0.06 g (16–18), while the amplitude distribution of maternal heartbeat and respiration is less than 0.015 g, and the amplitude of the signal generated by large maternal laughing, coughing, and body movement is greater than 0.1 g. As a result, the true fetal movement signal and the artifact signal can be distinguished by comparing the magnitude of the signal amplitude value.

Amplitude threshold pre-identification allows the signal which after pre-processing through the output, the signal greater than 0.1 g is directly judged as artifact noise, and the classified signal is non-fetal movement, by setting a limiter, when the upper and lower limits of the range of fetal movement at 0.015 g 0.06 g. If the signal amplitude distribution of one of the sensor’s axes is less than 0.015 g and the signal is between 0.06 g and 0.1 g, wait for the processing results of the remaining two axes of the accelerometer before proceeding to the next step of fetal movement identification.

### 3.3. Fetal movement recognition and classification unit

A complete feature dictionary is constructed by standard fetal movement signal training and dictionary learning, and the OMP algorithm is used to traverse the feature atoms in the dictionary and match the feature atoms with the pre-identified signal samples, calculate the reconstruction error of fetal movement signal samples on fetal movement and non-fetal movement feature based on sparse coefficients, and judge whether fetal movement occurs according to whether the error is reasonable or not. The specific steps of the fetal movement recognition algorithm are shown in Table 1.

**Table 1:** Fetal movement recognition algorithm.

**Algorithm**
**Input:** Pre-recognition signal Xi, i = 1, 2, …, 6. Feature dictionary Dk, k = 1,2. Sparsity coefficient T_0_.
**Output:** Recognition category Yi, i = 1, 2, …, 6. Initialize the parameters. **for** i = 1:6 **for** k = 1:2 **while** t < T_0_ Iteration number t = 1, reconstructed residual r_0_ = X_i_, set Λ_0_ = Ø, D=D_k_. Find the index λ_t_ where the reconstructed residual r_t-1_ matches the feature dictionary D, i.e., $\lambda_{t}=argmax_{j\in \Lambda_{t-1}}\;|r_{t-1},d_{j}|$ . Update the set of indexes Λ_t_ = Λ_t-1_∪λ_t_, reconstructing the dictionary D_t_ = [D_t-1_, d_λt_]. Compute using least squares to obtain ${}_{\hat{x}t}=\arg\underset{\hat{\hat{x}}}{\min}∥y-D\hat{x}{∥}_{2}^{2}$ . Update the reconstructed residuals $r_{t}=y-D_{t}{\hat{x}}_{t}$ . t = t + 1. **end while** Cache the sparse residuals e_k_ = r_t_. **end for** **if** e_1_ < e_2_, identify the signal as fetal Y_i_ = 1, **else**, identify the signal as non-fetal Y_i_ = 0. **end for**

## 4. Testing and analysis

### 4.1. Test solutions

The wearable fetal movement detection device test was carried out on four healthy primiparous pregnant volunteers, and the individual and fetal profiles of pregnant volunteers are shown in Table 2. All of whom were experiencing frequent and active fetal movement during their gestational cycle, and the average measurement recording time for each pregnant volunteer was 60 min. Because it is known from the literature (19–21) that the error rate of the fetal movement detection system in the sitting and standing positions of pregnant women is higher than in the lying position, this test was performed in the lying position. During the experiment, the experimental assistant assisted the pregnant volunteers in wearing the equipment and recorded the time when the pregnant women perceived fetal movement.

**Table 2:** The individual and fetal profiles of pregnant volunteers.

Pregnant volunteers	A	B	C	D
Age	27	26	27	28
Education level	Undergraduate	Undergraduate	Undergraduate	Undergraduate
Height/m	1.56	1.52	1.59	1.66
Weight/kg	61.8	63.4	62.5	65.3
Body mass index (BMI)	25.39	27.44	24.72	23.70
Health level	Wellness	Wellness	Wellness	Wellness
Number of fetuses	1	1	1	1
Gestational age	32 week + 4	32 week + 5	32 week + 4	32 week + 6
Amniotic fluid index (AFI)/mm	136	153	145	121

**Figure 6:**
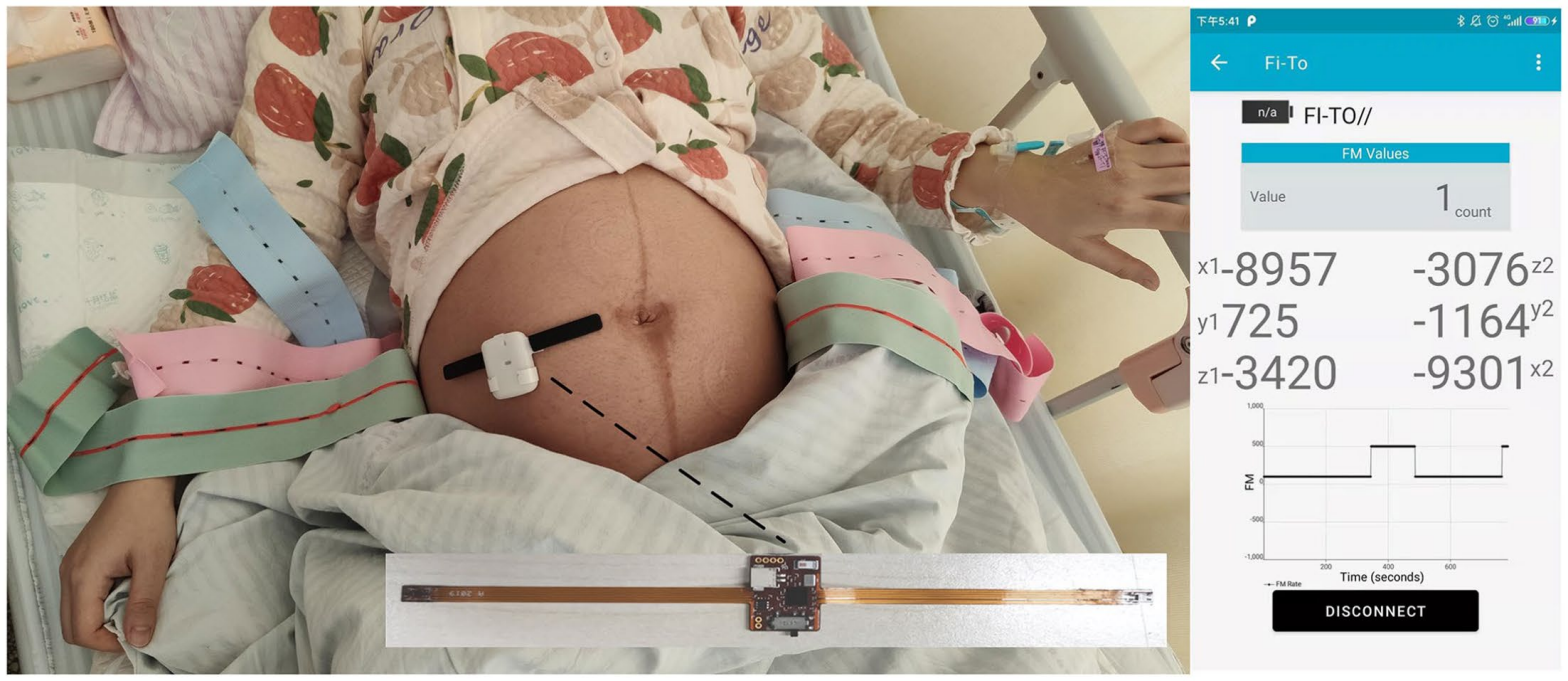
System equipment and test scenario diagram.

The sensor was fixed on the area of the pregnant woman’s abdomen where the fetal movement was most strongly perceived using a fetal monitoring belt during the fetal movement data sampling (22–24), and the sensor would collect the change of acceleration value as the fetus moved, and then transmit the collected sensor acceleration value to the main controller through the IIC communication interface. Before beginning the test, the pregnant woman recalls and locates the area in her abdomen with the strongest perception of fetal movement, and the host device is placed directly above the area and secured with the belt so that the sensors on both sides of the device can collect the important raw signals, as shown in Figure 6.

The real fetal movements actively perceived by pregnant women are used as the standard in this test protocol, and the recognition rate (True detection rate, TDR) and the correct rate (Precision, PPV) are used as performance indicators for evaluating this fetal movement detection device (18, 25, 26). The TDR represents the ratio of correctly recognized samples to total number of samples and is expressed as Equation (4).


(4) TDR=EN/TN×100%

Where EN is the number of valid fetal movements identified by the fetal movement collection system and TN is the number of real fetal movements actively perceived by pregnant women. Equation (5) is the formula for the correct rate PPV, which indicates the ratio of the number of correctly identified positive samples to the number of samples marked as positive in the identification result, where FN denotes the number of false identifications.


(5) PPV=EN/(TN+FN)×100%

### 4.2. Results analysis

**Figure 7:**
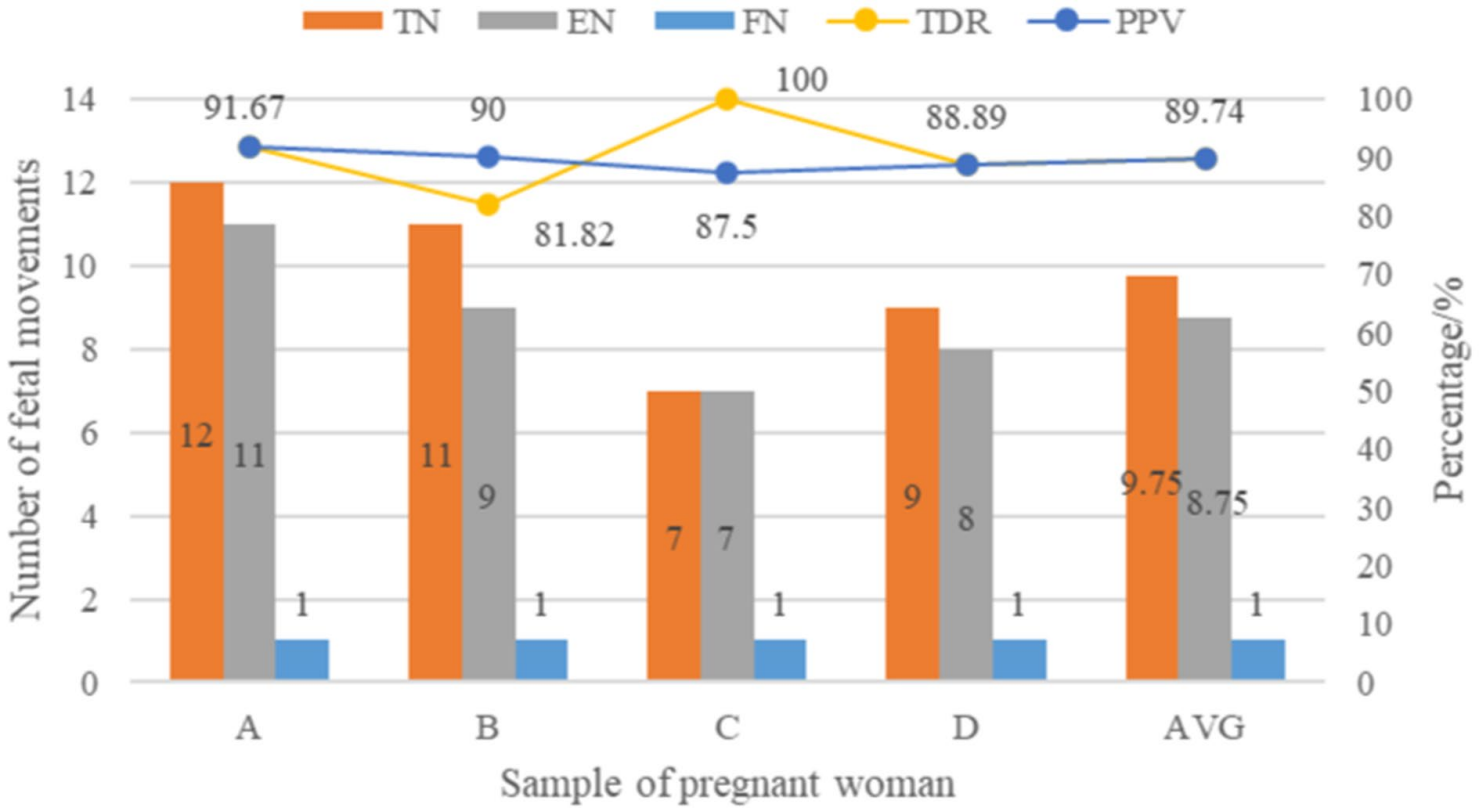
Comparison of experimental data of fetal movement detection system.

Figure 7 depicts a comparison of test data from this fetal movement detection system host device on four pregnant volunteers, as per the test protocol. The first volunteer A perceived 12 real fetal movements, the system recognized 11 fetal movements and 1 false recognition, and the recognition rate and correct rate of the system were both 91.67%. Volunteer B perceived 11 real fetal movements, the system recognized 9 fetal movements and 1 false recognition, with a recognition rate of 81.82% and a correct rate of 90%. Volunteer C perceived 7 real fetal movements, and the system recognized 7 fetal movements and 1 false recognition, with a recognition rate of 100% and a correct rate of 87.5%. The fourth volunteer D perceived 9 real fetal movements, the system recognized 8 fetal movements and 1 false recognition, and the recognition rate and correct rate were both 88.89%.

After summarizing and averaging the results of the above volunteers’ samples, it was found that the average recognition rate and correct rate of the fetal movement detection system in recognizing fetal movement signals were 89.74%. Furthermore, the presence of false recognition by the system may be caused by pregnant women’s random limb movement during the measurement process, which generates artifact signals with high similarity to the time-frequency characteristics of fetal movement signals. The active perception of pregnant women self-counting fetal movements has no effect on pregnant women or fetuses (27, 28), but the method is susceptible to many factors such as the surrounding environment and personal emotions (29–31), and the short-term detection data is highly reliable. At the same time, some weak fetal movement signals may not be fully captured by the acceleration sensor mounted on the pregnant woman’s abdomen, resulting in a low real detection recognition rate.

## 5. Conclusion

The fetal movement detection device is becoming more affordable, practical, and wearable as micro-embedded technology advances. This paper proposes a fetal movement detection system for pregnant women that consists of two acceleration sensors, a Cortex-M4 main control chip, battery management, a Bluetooth antenna, and a smartphone, as well as other functional modules. The wearable fetal movement detection system’s structure, the accelerometer application circuit, and the Cortex-M4 main control chip application circuit are all described in detail. The overall system software design unit, sensor data collection program design unit, fetal movement recognition and algorithm design unit, and Bluetooth data communication unit are all investigated in the system software and algorithm design scheme. Finally, the system testing scheme is presented, and the test results are examined. Four healthy pregnant volunteers in active fetal movement gestational cycle were used as test subjects based on real fetal movement actively perceived by pregnant women within 60 min. The average recognition rate and correct rate of fetal movement signal detection of this system were 89.74% based on system testing and comparison analysis, indicating that the system detection accuracy is high and can be used in the field of fetal health monitoring.

The wearable fetal movement detection system is designed to collect fetal movement signals in real-time and display and store the results in real-time in the monitoring APP on the cell phone, allowing for long-term harmless fetal movement detection, ensuring the healthy growth of the fetus, and relieving the strain on medical resources. Fetal movement monitoring and recognition is a highly technical task that involves interdisciplinary study areas and calls for the use of both clinical medical expertise and artificial intelligence tools. We have completed proof-of-principle experiments and obtained some study findings at this point. Only four pregnant volunteers have been validated thus far, therefore the sample data obtained and examined are on the low side and will need to be improved upon in the future. Fetal movement is a direct form of expression used to detect the health of the fetus in the mother’s womb, but it is easily affected by the mother’s behavior and external factors, and the accuracy of fetal movement recognition can be improved further in the future through algorithm improvement and optimization. For instance, standard biomedical fetal movement databases are created, several sensors are employed to create detection arrays, full and highly generalizable recognition models are investigated, etc.

## Data availability statement

The original contributions presented in the study are included in the article/supplementary material, further inquiries can be directed to the corresponding author.

## Author contributions

YX, MQ, and TS contributed to conception and design of the study. MQ and YX organized the methodology and validation. YL and YX organized data curation and performed analysis. MQ and YX wrote the first draft of the manuscript. YL and TS wrote sections of the manuscript. All authors contributed to manuscript revision, read, and approved the submitted version.

## Funding

This research was funded by the Research Project for Young and Middle-aged Teachers in Guangxi Universities under Grant No: 2021KY0617, Special Research Project of Hechi University under Grant No: 2022YLXK003. This research was financially supported by First-class Discipline Construction Project of Hechi University, Guangxi Colleges and Universities Key Laboratory of AI and Information Processing (Hechi University), Education Department of Guangxi Zhuang Autonomous Region.

## Conflict of interest

The authors declare that the research was conducted in the absence of any commercial or financial relationships that could be construed as a potential conflict of interest.

## Publisher’s note

All claims expressed in this article are solely those of the authors and do not necessarily represent those of their affiliated organizations, or those of the publisher, the editors and the reviewers. Any product that may be evaluated in this article, or claim that may be made by its manufacturer, is not guaranteed or endorsed by the publisher.
